# Sequential fractionation of the lignocellulosic components in hardwood based on steam explosion and hydrotropic extraction

**DOI:** 10.1186/s13068-018-1346-y

**Published:** 2019-01-04

**Authors:** Johanna Olsson, Vera Novy, Fredrik Nielsen, Ola Wallberg, Mats Galbe

**Affiliations:** Department of Chemical Engineering, Lund University, P.O. Box 124, 221 00 Lund, Sweden

**Keywords:** Steam pretreatment, Hydrotrope, Hardwood, Lignin extraction, Biorefinery, Lignocellulose, Enzymatic hydrolysis, Sodium xylene sulfonate

## Abstract

**Background:**

The forest biorefinery plays an important part in the evolving circular bioeconomy due to its capacity to produce a portfolio of bio-based and sustainable fuels, chemicals, and materials. To tap into its true potential, more efficient and environmentally benign methods are needed to fractionate woody biomass into its main components (cellulose, hemicellulose, and lignin) without reducing their potential for valorization. This work presents a sequential fractionation method for hardwood based on steam pretreatment (STEX) and hydrotropic extraction (HEX) with sodium xylene sulfonate. By prehydrolyzing the hemicellulose (STEX) and subsequently extract the lignin from the cellulose fraction (HEX), the major wood components can be recovered in separate process streams and be further valorized.

**Results:**

Using autocatalyzed STEX and HEX, hemicellulose (> 70%) and lignin (~ 50%) were successfully fractionated and recovered in separate liquid streams and cellulose preserved (99%) and enriched (~ twofold) in the retained solids. Investigation of pretreatment conditions during HEX showed only incremental effects of temperature (150–190 °C) and hold-up time (2–8 h) variations on the fractionation efficiency. The hydrolyzability of the cellulose-rich solids was analyzed and showed higher cellulose conversion when treated with the combined process (47%) than with HEX alone (29%), but was inferior to STEX alone (75%). Protein adsorption and surface structure analysis suggested decreased accessibility due to the collapse of the fibrillose cellulose structure and an increasingly hydrophobic lignin as potential reasons.

**Conclusion:**

This work shows the potential of sequential STEX and HEX to fractionate and isolate cellulose, hemicellulose, and a sulfur-free lignin in separate product streams, in an efficient, sustainable, and scalable process.

**Electronic supplementary material:**

The online version of this article (10.1186/s13068-018-1346-y) contains supplementary material, which is available to authorized users.

## Background

Wood is a valuable raw material for a wide variety of applications, ranging from the use of timber for construction to the production of novel high-end cellulose hydrogels for the cosmetic and pharmaceutical industries [1, 2]. Certain applications thereby require intact wood, with all its components—cellulose, hemicellulose, and lignin—and their complex interactions. Others require individual components, which can be obtained by fractionation of the wood. One application with fractionation at scale is the pulp and paper industry, a sector that is producing a portfolio of renewable products from the single constituents of woody biomass. In response to the decreasing demand for paper and board products, many pulp mills are being repurposed to create new and complementary revenue streams from novel products and markets. A current trend is retrofitting of Kraft pulping capacity to produce dissolving-grade pulp from hardwood [3]. In the prehydrolysis-Kraft process, a prehydrolysis step extracts the hemicelluloses prior to Kraft pulping [3]. Apart from dissolving pulp, the value proposition of the process includes valorization of the hemicellulosic monomers and oligomers that are recovered from the prehydrolysis liquid [4] and Kraft lignin [5, 6]. However, covalently bonded sulfur in lignin represents a major drawback, because it hinders the catalytic upgrading and refining of lignin [7]. Sulfur-free processes, such as alkali-cooking using anthraquinone [8], can yield the desired delignification; however, the toxicity of anthraquinone limits its commercial potential and raises considerable environmental concerns [9].

Steam pretreatment, often referred to as steam explosion (STEX), has been examined extensively as a biomass fractionation method [10–12]. STEX results in the hydrolysis of glycosidic bonds in the hemicelluloses and, to a lesser extent, in the cellulose [13], hence solubilizing the hemicellulose while preserving the cellulose in the woody biomass. The hemicellulosic sugars can then be recovered in the liquid fraction. In this regard, STEX shares most features with the aqueous auto-hydrolysis, which is commonly practiced in the dissolving pulp industry [5]. By disrupting the lignin–carbohydrate complexes (LCC), STEX further increases the accessibility of cellulose to enzymes and proteins and can thus enhance the hydrolyzability of the substrate [14, 15].

However, more recalcitrant biomass requires the STEX process to be operated under high severity conditions. This can lead to secondary degradation reactions, which reduce the hemicellulose yield and result in the formation of inhibitory compounds with adverse effects on subsequent bioconversion steps [16, 17]. High severity conditions can also degrade and modify the chemical structure of the lignin and compromise its yield [14, 15, 18]. The competing de- and re-polymerization reactions generate a lignin with increased prevalence of condensed structures and less reactivity and solubility [18], limiting its potential for valorization. To avoid excessive degradation of hemicellulose and lignin, multistep pretreatment processes have been described, including the sequential solubilization of components by performing STEX under various process conditions [15, 19, 20] or by combining it with other methods [21, 22].

Other pretreatment methods have been developed to selectively extract lignin from woody biomass. These include organosolv, alkali treatments, deep eutectic solvents, ionic liquids, and hydrotropic extraction (HEX) [23–25]. HEX has gained increasing attention as a green biorefinery technology, due to its efficient delignification of woody [26, 27] and non-woody biomass [28–30], its neutral pH conditions, the relative ease with which sulfur-free lignin is recovered, and the possibility of reusing the hydrotrope [31, 32].

Hydrotropes are amphiphilic organic salts that increase the solubility of otherwise insoluble, or sparingly soluble, organic compounds in aqueous solutions [33, 34]. Frequently used hydrotropic agents for extracting lignin from woody biomass include SXS [26, 35], alkylbenzenesulfonate [32, 36], and sodium cumene sulfonate [37, 38], where SXS has emerged as most promising candidate due to its high efficiency and low cost [31]. HEX of woody biomass has typically been performed with SXS concentrations of 30–40 wt%, liquid-to-wood ratios between 4 and 8, temperatures of 130 to 170 °C, and hold-up times of 0.5 to 12 h, with [26, 39, 40] and without additives [35, 41–43]. Lignin yields are generally higher with hardwood than with softwood substrates [44], and HEX with SXS is more efficient in extracting syringyl (S) than guaiacyl (G) lignin moieties [43]. With hardwood substrates, up to 70% of the lignin has been extracted with SXS [35], wherein HEX was particularly efficient in removing surface lignin [43]. The lignin recovered after HEX typically maintains the monolignol ratios of the raw material, its elemental composition resembles that of a technical lignin (higher carbon content and lower oxygen and hydrogen levels than the original lignin), and the reactivity is comparable with that of Alcell organosolv lignin [39]. HEX-derived lignin contains few non-lignin contaminants [39] and can be virtually sulfur-free [32, 35, 39].

Overall, HEX is a promising and green method for lignin extraction. However, when used as a single-step pretreatment, the recovery of hemicellulose is compromised [43, 45].

The aim of this study was to establish an environmentally benign fractionation method that omits the use of sulfur, separates the lignocellulosic components efficiently. Here, we present a sequential fractionation process for hardwood, in which birch and beech wood chips were first treated with STEX to solubilize hemicellulose. Lignin was subsequently extracted from the residual solids by HEX, resulting in a cellulose-rich solid fraction. The effects of HEX process parameters (temperature and hold-up time) on the fractionation efficiency were evaluated, a mass balance-based process analysis performed, and the hydrolyzability of the cellulose-enriched solids assessed.

## Methods

### Raw material

The raw material was a mixture of 80% birch (*Betula pendula*) and 20% European beech (*Fagus sylvatica*) wood chips, provided by Södra Cell (Mörrum, Sweden). The composition of the raw material mixture, SF_raw mat_, is presented in Table 1. The wood chips were size reduced using a knife mill (Retsch GmbH, Germany) that was fitted with a 20-mm screen and sieved to retrieve the 2–10-mm fraction.

**Table 1:** Composition of the raw material and solid and liquid fractions after STEX pretreatment

	Raw material	HEX-treated material		STEX treated material	
	SF_raw mat_ (wt%)	SF_HEX_ (wt%)	LF_HEX_ (g/L)	SF_STEX_ (wt%)	LF_STEX_ (g/L)
Carbohydrates, *thereof*	67.6 ± 1.4	83.9 ± 0.1	na	67.1 ± 1.1	na
Glucan/glucose	39.4 ± 0.7	67.1 ± 0.1	0.4 ± 0.0	59.2 ± 0.7	2.6 ± 0.0
Xylan/xylose	22.2 ± 0.5	13.2 ± 0.0	10.1 ± 0.1	5.7 ± 0.0	43.4 ± 0.7
Arabinan/arabinose	0.6 ± 0.0	BDL	0.2 ± 0.0	BDL	0.7 ± 0.0
Galactan/galactose	1.8 ± 0.1	BDL	0.7 ± 0.0	BDL	2.2 ± 0.0
Mannan/mannose	3.7 ± 0.2	3.6 ± 0.0	0.5 ± 0.0	1.9 ± 0.0	5.7 ± 0.2
Lignin, thereof	27.2 ± 1.1	14.5 ± 0.1	na	30.4 ± 0.0	na
Acid-soluble	6.5 ± 0.0	3.4 ± 0.2	na	2.9 ± 0.1	na
Acid-insoluble	20.7 ± 1.0	11.1 ± 0.1	na	27.5 ± 0.1	na
Ash	BDL	BDL	na	BDL	na
Recovery	94.9 ± 2.5	98.4 ± 0.0	na	97.6 ± 1.1	na

Data represent mean values and standard deviation. Analyses were performed in duplicate

*BDL* below detection limit, *na* not applicable, *SF* solid fraction, *LF* liquid fraction, *STEX* steam explosion, *HEX* hydrotropic extraction

### Two-step pretreatment method

The pretreatment method comprised two sequential steps: autocatalyzed steam explosion (STEX), followed by hydrotropic extraction (HEX). For comparison, STEX and HEX were also performed separately.

#### Steam explosion (STEX)

Milled wood chips were impregnated with water overnight at room temperature, yielding a dry matter content (DM) of approximately 50 wt%. The impregnated feedstock was treated with STEX in batches of 750 g DM at 210 °C for 5 min in a preheated 10 L pretreatment reactor. The pretreatment reactor has been described previously [46]. The STEX conditions were chosen based on earlier studies on STEX pretreatment of hardwood [15, 47, 48]. The severity factor, S_0_, of the STEX conditions was 4.2, calculated according to [49]. After STEX, the liquid and solid fractions were separated by filtration using a hydraulic filter press (HP5M, Fischer Maschinenfabrik, Germany) at six bar. The compositions of the solid fraction, SF_STEX_, and liquid fraction, LF_STEX_, are summarized in Table 1. Prior to HEX, the SF_STEX_ was washed by suspending 1 kg of wet material in 10 L water, soaked for 1 h with agitation, and dewatered by filtration using a hydraulic filter press at 6 bar. The DM content of SF_STEX_ was approximately 35 wt%.

#### Hydrotropic extraction (HEX)

HEX was performed in a 2 L stirred tank reactor (Polyclave, Büchi AG, Switzerland) that was equipped with a stirrer unit (Cyclone 300, Büchi AG) and a thermostat (Unistat T305, Huber Kältenmaschinenbau AG, Germany). The hydrotropic solution contained 40% (w/v) SXS (Stepanate SXS-93, Alsiano, Denmark) in aqueous solution. For the HEX of prehydrolyzed material, 140 g of wet SF_STEX_ (~ 50 g dry) was mixed with 1050 g SXS solution and loaded to the reactor. The extraction was run isothermally under constant agitation at 350 rpm and under 2 different process conditions: (i) 150 °C for 8 h and (ii) 190 °C for 4 h. The resulting solid and liquid phases were denoted SF_STEX+HEX150/8_, SF_STEX+HEX190/4_ and LF_STEX+HEX150/8_, and LF_STEX+HEX190/4_, respectively. The effects of process parameters on extraction efficiency were further assessed by varying the temperature (150, 170, 180, and 190 °C, with a constant hold-up time of 4 h) or hold-up time (0.5, 2, 4, 6, and 8 h, at a constant temperature of 150 °C). After HEX, the solid phase was separated from the liquid phase by filtration using a hydraulic filter press. HEX was also performed using SF_raw mat_ as feedstock. The process conditions were 150 °C and 8 h using the protocol above. The resulting liquid and solid phases are denoted LF_HEX150/8_ and SF_HEX150/8_, respectively.

### Enzymatic hydrolysis

The hydrolyzability of SF_STEX_, SF_HEX150/8_, SF_STEX+HEX150/8_, and SF_STEX+HEX190/4_ was measured in triplicates in 250-mL screw cap bottles using the Cellic Ctec2 commercial enzyme mixture (Novozymes, Denmark). Prior to the enzymatic hydrolysis, all substrates were washed with water and 0.1 M sodium acetate buffer, pH 4.8 (Na–Ac). The substrates were then suspended in Na–Ac, yielding a substrate loading of 3 wt%. The substrate loading was chosen to allow kinetic analysis of the hydrolysis, without limiting the reaction by high-solid loadings related issues, e.g., mass and heat transfer. The total working weight was 25 g. The substrate suspension was autoclaved at 121 °C for 20 min, after which the sterile filtrated enzyme solution was added aseptically. Two enzyme loadings were applied: 10 and 20 FPU/g cellulose. The reactions were performed at 50 °C for 72 h in an orbital shaker (Lab-Therm, Kühner AG, Switzerland) with a constant agitation at 200 rpm. Samples were taken from the hydrolysis experiments at regular intervals. Immediate sample workup comprised deactivation of the enzymes at 100 °C for 10 min, centrifugation for 5 min at 13,000 rpm, and filtration of the supernatant (0.22-μm filters). The samples were stored at 4 °C until the carbohydrate analysis.

The conversion of cellulose to glucose, *Y*_g_ [% of theoretical maximum], was calculated by the equation below, which provide sufficient accuracy of the yield prediction at low solids loadings [50]:

$$Y_{\text{g}} = {{\left( {C_{\text{g}} - C_{{{\text{g}}0}} } \right)} \mathord{\left/ {\vphantom {{\left( {C_{\text{g}} - C_{{{\text{g}}0}} } \right)} {\left( {\varphi_{\text{G}} \cdot C_{\text{is0}} \cdot x_{\text{G0}} } \right)}}} \right. \kern-0pt} {\left( {\varphi_{\text{G}} \cdot C_{\text{is0}} \cdot x_{\text{G0}} } \right)}} \cdot 100,$$

where *C*_g_ [g/L] is the concentration of solubilized glucose in the sample supernatant, *C*_g0_ [g/L] is the initial glucose concentration, *φ*_G_ [−] is the molecular weight ratio of glucose-to-glucan monomer (*φ*_G_= 180/162 = 1.11), *C*_is0_ [g/L] is the initial concentration of insoluble solids, and *x*_G0_ [−] is the initial mass fraction of glucan in the insoluble solids.

### Protein adsorption experiments

The adsorption of proteins onto SF_STEX_, SF_STEX+HEX150/8_, and SF_STEX+HEX190/4_ was analyzed in 2 mL Eppendorf tubes with a substrate loading of 3 wt% in Na–Ac and a total reaction weight of 1.8 g. Cellic Ctec2 was used to analyze enzyme–substrate binding with enzyme loadings of 10 and 20 FPU/g cellulose. Bovine serum albumin (BSA) was included to analyze nonspecific protein–substrate adsorption. BSA concentrations were equal to the total protein concentration in the Cellic Ctec2 experiments, which had a specific activity of 2.06 FPU/mg_Protein_.

All preparations were incubated at 4 °C for 4 h with constant agitation at 200 rpm on an orbital shaker. Next, the reaction mixture was centrifuged (13,000 rpm, 5 min), and the concentration of unbound proteins in the supernatant was measured. For this, the proteins were first precipitated with 40 µL 500 mM KH_2_PO_4_ (pH 7.4) and 40 µL 250 mM CaCl_2_ and then harvested by centrifugation. After the pellet was washed thoroughly with absolute ethanol, it was resuspended in 2 mL ready-to-use Bradford reagent (Sigma Aldrich, USA). The absorption was measured at 595 nm, and the protein concentration was determined against an external BSA standard.

### Scanning electron microscope (SEM) imaging

Scanning electron microscope (SEM) images of SF_STEX_, SF_STEX+HEX150/8_, and SF_STEX+HEX190/4_ were taken using a JSM-6700F SEM (JEOL, Japan), which was set to an acceleration voltage of 10 kV. The samples were prepared by depositing oven-dry material on a brass stub, to which they were fixated with double-sided tape and covered with a 15-nm layer of Au/Pd using a sputter coater (SCD00, Oerlikon Balzers AG, Liechtenstein).

### Analytical procedures

Dry matter (DM) content is defined as the oven-dry weight at 105 °C and was measured in triplicate using a National Renewable Energy Laboratory (NREL) standard method [51]. The composition of structural carbohydrates and lignin in the solids [52] and the sugars and degradation products in the liquid process streams [53] were analyzed by NREL standard methods. The HEX-treated materials were washed in a three-step procedure with 0.1 M NaOH, 0.05 M NaOH, and water prior to analysis to remove the hydrotropic agent and prevent redeposition of lignin onto the solids. The wavelength and extinction coefficient for the measurement and quantification of acid-soluble lignin was 240 nm and 25 L/g/cm, respectively [52]. All analyses were performed in triplicates.

Carbohydrates were measured by isocratic high-performance anion-exchange chromatography with pulsed amperometric detection, using an ICS-3000 chromatography system (Dionex, USA) that was equipped with a Carbo Pac PA1 analytical column (Dionex, USA). Measurements were performed at 30 °C using deionized water as the eluent at a flow rate of 1 mL/min.

### Mass balance-based process analysis

**Fig. 1:**
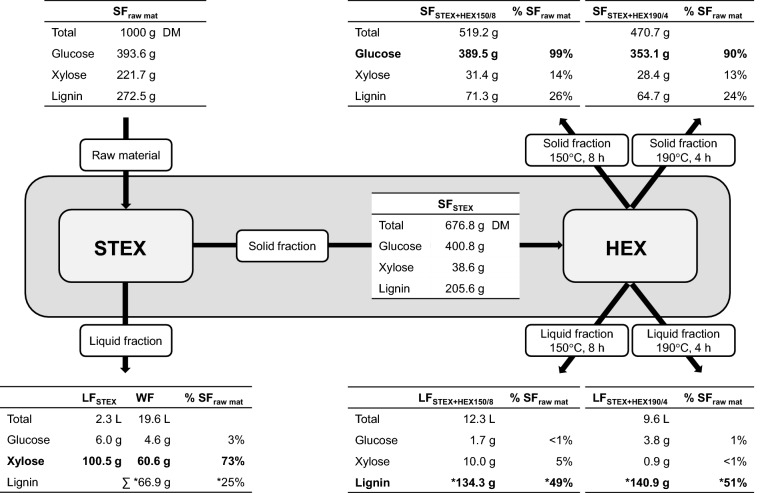
Mass balance-based process analysis of the two-step pretreatment process, combining STEX with HEX150/8 and HEX190/4, respectively. The data represent the composition of the liquid and solid fractions, as presented in Tables 1 and 2. Process streams were determined by measuring the weight and volume of all processing steps, including the wash fractions (WF) after STEX. The numbers marked with an asterisk were not measured but determined based on mass balances. Note: the rescaling of the masses and volumes to 1000 g of raw material led to minor over and underestimation of component masses, resulting in mass balances that are not fully closed (i.e., glucose in SF_raw mat_ and SF_STEX_)

Mass balance-based analysis of the pretreatment process (Fig. 1) was performed, for which the mass, volume, and carbohydrate composition of all process streams, including the wash fractions, were measured and recorded.

## Results and discussion

### Fractionation of hardwood by STEX and HEX: analysis and comparison of materials treated under various conditions

This study examined three methods for fractionating hardwood: hydrotropic extraction (HEX alone), steam explosion (STEX alone), and the combination thereof (STEX + HEX). In the following, the composition of the resulting process streams from various treatment conditions is presented and discussed.

#### HEX-alone treatment of hardwood

Hardwood chips were treated with HEX as a freestanding process, constituting a base case and benchmark values for comparison. Following literature recommendations [35, 41], a 40% aqueous SXS was used to extract lignin at 150 °C for 8 h. The compositions of the product streams after HEX are presented in Table 1.

The relative amount of total structural carbohydrates in the biomass increased from 68 to 84 wt% by HEX—nearly, a 1.25-fold increase. The relative glucan content rose from 39 to 67 wt%, primarily due to the removal of lignin and xylan, the levels of which decreased from 27 to 14 wt% and 22 to 13 wt%, respectively (Table 1). Of all hemicellulosic sugars, the solid and liquid fractions contained mainly xylose (Tables 1 and 2). The other hemicellulosic sugars (i.e., arabinose, mannose, and galactose) were present at low or trace amounts, prompting us to use xylose as proxy for the hemicellulose component hereinafter.

**Table 2:** Composition of the solid and liquid fractions of the STEX + HEX-pretreated materials, HEX treated at 150 °C for 8 h (STEX + HEX-150/8) and at 190 °C for 4 h (STEX + HEX-190/4)

	Standard conditions		Modified conditions	
	SF_STEX+HEX150/8_ (wt%)	LF_STEX+HEX150/8_ (g/L)	SF_STEX+HEX190/4_ (wt%)	LF_STEX+HEX190/4_ (g/L)
Carbohydrates, *thereof*	84.1 ± 2.2	na	83.9 ± 4.2	na
Glucan/glucose	75.0 ± 1.3	0.1 ± 0.0	77.6 ± 2.7	0.2 ± 0.0
Xylan/xylose	6.0 ± 0.1	0.8 ± 0.0	3.6 ± 0.1	3.2 ± 0.0
Arabinan/arabinose	BDL	BDL	BDL	BDL
Galactan/galactose	BDL	0.1 ± 0.0	BDL	0.2 ± 0.0
Mannan/mannose	3.0 ± 0.9	0.2 ± 0.0	2.7 ± 1.4	0.4 ± 0.0
Lignin, thereof	13.7 ± 0.6	na	13.5 ± 0.2	na
Acid-soluble	2.2 ± 0.2	na	1.6 ± 0.1	na
Acid-insoluble	11.6 ± 0.4	na	11.5 ± 0.0	na
Ash	BDL	na	BDL	na
Recovery	97.8 ± 2.2	na	97.4 ± 4.0	na

Data represent mean values and standard deviation. Analyses were performed in duplicate

*BDL* below detection limit, *na* not applicable, *SF* solid fraction, *LF* liquid fraction, *STEX* steam explosion, *HEX* hydrotropic extraction

The HEX-alone treatment resulted in 72 wt% lignin removal, extraction of 68 wt% of the xylose, and preservation of most of the cellulose (> 91 wt%). This is par with the 70.1 wt% lignin removal that was obtained with SXS treatment of birch chips [35]. Considering the proposed impact of the lignin chemistry, lignin topochemistry, and overall wood structure on the HEX process [35], it is surprising that species-dependent variations did not result in greater differences in lignin removal efficiency.

#### STEX as a prehydrolysis step to HEX

As shown in Table 1, a significant fraction of the hemicellulose was extracted with the lignin during HEX. The hemicellulose represents an impurity in the cellulose and lignin process streams, necessitating further fractionation. Although the use of additives in HEX has been shown to enhance the extraction of hemicellulose [26, 39, 40], they typically increase cellulose hydrolysis and the competing lignin condensation reactions, limiting the recovery of cellulose and the removal of lignin. Hemicellulose further cannot easily be separated from the hydrotrope after precipitation of the lignin, which complicates the recycling of the hydrotrope and compromises the value proposition of the process. Instead, we propose a two-step process, STEX followed by HEX, to selectively separate and recover them in individual product streams.

Using STEX as a prehydrolysis step, the relative xylan content of the material decreased from 22 (SF_raw material_) to 6 wt% (SF_STEX_) while preserving the cellulose (Table 1 and Fig. 1). Over 80 wt% of the total xylan content and 25 wt% of the lignin in the raw material were removed (Fig. 1). The relative cellulose content in the material rose from 39 (SF_raw material_) to 59 wt% (SF_STEX_). Hemicellulose removal and recovery can be enhanced using an acid catalyst in the STEX [22]. However, additives can compromise the recovery of cellulose, increase the solubilization and condensation of lignin, and, depending on the choice of acid, and result in contamination of the lignin with sulfur [22].

HEX was used for the selective extraction of lignin from SF_STEX_. The same temperature and hold-up time were applied in the combined process as in the HEX-alone approach (150 °C and 8 h). With the combined process, the relative glucan content of the material was increased to 75 wt% (Table 2), which is 1.12-fold higher compared with HEX alone. The combined process resulted in 74 wt% of lignin removal.

To enhance the removal of lignin during HEX, the extraction temperature was increased to 190 °C. A positive correlation between lignin removal and extraction temperature has been suggested previously for HEX of sugar cane [32] and hybrid Eucalyptus [42] using SXS. Due to the marginal effect of increases in hold-up time on the extraction efficiency after a certain stage [32], as we will also demonstrate below, the hold-up time was limited to 4 h at the higher temperature. The results from the combined process under the modified conditions (STEX + HEX190/4) are summarized in Table 2. The relative cellulose content in the material (SF_STEX+HEX190/4_) was enriched to 78 wt%, and the xylan content decreased to 4 wt%. The relative lignin content declined to 13.7 wt% in SF_STEX+HEX190/4_, which is at par with the lignin content in SF_STEX+HEX150/8_ (13.5 wt%) and SF_HEX150/8_ (14.5 wt%).

Similar to the relative lignin content, the removal of lignin in the HEX step was decreased incrementally from 72 wt% for HEX150/8 to 65 wt% for STEX + HEX150/8 and 69 wt% for STEX + HEX190/4. Likely reasons are changes in lignin abundancy, morphology, and chemistry as a result of STEX [14, 18], which can lower the efficiency of the extraction. In particular, competing lignin re-polymerization reactions are expected to predominate under the STEX pretreatment conditions that were used (severity factor, *S*_0_ = 4.2), thus causing redeposition of lignin onto the solids [18].

#### Mass-balanced process analysis

To evaluate the efficiency of the two main processes for fractionating wood and recovering xylose, lignin, and cellulose in separate process streams, a mass balance-based process analysis was performed. The results are presented in Fig. 1.

STEX prehydrolysis resulted in the removal of 83 wt% of the total xylose content in the raw material, 88 wt% of which could be recovered in the liquid process streams after STEX (LF_STEX_ and WF; Table 1 and Fig. 1). The xylose that is recovered is a valuable carbon source for biological conversions—for example, with pentose-fermenting yeast and biocatalysts [4, 54]. The main impurity in the hemicellulose product stream was the lignin that was solubilized by STEX (Fig. 1).

Independent of the process conditions, approximately 50% of the lignin could be recovered from the liquid process stream after HEX (LF_STEX+HEX190/4_ and LF_STEX+HEX150/8_, Fig. 1). The recovery strategy—comprising precipitation of lignin by diluting the SXS below the MHC with water, filtration of the lignin precipitate, and SXS recovery by evaporation—has been shown to generate a highly pure lignin and a recyclable SXS [26, 41]. The properties of the lignin are expected to be similar to those of a typical technical lignin. However, because autocatalyzed STEX does not involve sulfur and because the SXS does not bind covalently to the lignocellulosic components, an obstacle to catalytic refining and upgrading of the lignin may be circumvented [6, 7].

The cellulose content of the solid fraction after the two-step process increased 1.12- (STEX + HEX150/8) to 1.16-fold (STEX + HEX190/4) compared with the HEX-alone process. The cellulose content in SF_STEX+HEX190/4_ (78%) was slightly higher as compared to SF_STEX+HEX150/8_ (75%). However, the cellulose component was preserved with STEX + HEX150/8 (99 wt% recovery), whereas the incremental improvement with STEX + HEX190/4 was achieved at the expense of the recovery of cellulose (90 wt%, Fig. 1). The cellulose-enriched product stream can be used as feedstock for further refining to dissolving-grade pulp (typically > 90 wt% cellulose content, < 5 wt% hemicellulose, < 0.05 wt% lignin, and application-specific quality demands [55]), which is typically produced at yields of 30–38% in conventional processes [5, 55]. Alternatively, the cellulose-enriched solids can be deconstructed enzymatically to a meet the requirements for further valorization.

#### The influence of temperature and hold-up time during HEX on the composition of pretreated materials

**Fig. 2:**
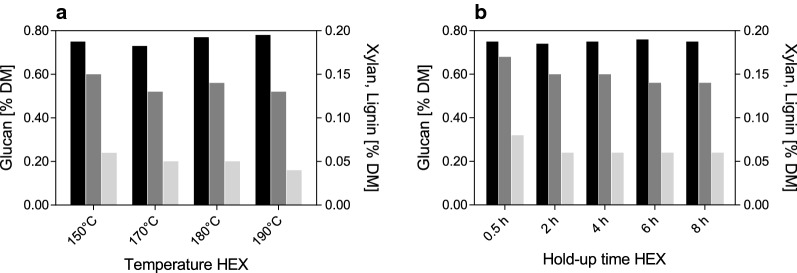
Influence of temperature (panel a) and hold-up time (panel b) during HEX on the composition of the solid fraction. Glucan (black), lignin (dark grey), and xylan (light grey) are shown as percentage dry mass. All materials were STEX-pretreated prior to HEX

Based on the low compositional variation of SF_STEX+HEX150/8_ and SF_STEX+HEX190/4_, the impact of temperature and hold-up time was analyzed in more detail. Figure 2a shows the effect of temperature on the extraction efficiency, during which the hold-up time was kept constant (150–190 °C for 4 h). Although a positive correlation between lignin removal and temperature has been suggested previously [32, 42], only incremental improvements were obtained in this study. Thus, the relative glucan and lignin content were 73–78 wt% and 13–15 wt%, respectively, under all conditions. The relative xylan content decreased from 6 wt% to less than 4 wt% as the temperature rose from 150 °C to 190 °C.

The effect of hold-up time during HEX (0.5–8 h at 150 °C) on the composition of the solid fraction is presented in Fig. 2b. Here, the relative glucan content varied between 74 and 76 wt%. The relative xylan content remained constant at approximately 6 wt% from 2 h onward, and the lignin content fell slightly between 2 h (15 wt%) and 8 h (14 wt%). Similar improvements in lignin removal with increasing hold-up time have been reported previously for HEX with SXS [32]. These results demonstrate that the hold-up time can be shortened to at least 2 h without any significant loss of delignification, rendering the process more efficient.

The reason for the unexpectedly low lignin removal efficiency with increasing hold-up time (> 2 h) and temperatures (150–190 °C) can probably be attributed to the structural and chemical changes that occur during STEX. Disruption of the biomass structure and redistribution of lignin increase the fraction of surface lignin that is easily accessible, facilitating rapid extraction of lignin [43]. However, the structural changes during STEX might also impede the penetration of SXS due to mass transfer resistance. The latter has been described as major factor affecting the efficiency of HEX [32], and might thus limit the removal of non-surface lignin in STEX-pretreated material.

### Analysis of the hydrolyzability of STEX-, HEX-, and STEX + HEX-pretreated materials

The purpose of the pretreatment is to fractionate the biomass and prime it for further valorization, e.g., saccharification of the cellulose to a sugar platform. Thus, the effects of the pretreatment conditions (HEX150/8, STEX, STEX + HEX150/8, and STEX + HEX190/4) on the enzymatic hydrolyzability of the cellulose-enriched fractions were examined.

#### The hydrolyzability of SF_HEX150/8_

First, the hydrolyzability of SF_HEX150/8_ was analyzed. The conversion yields and the initial rates of the hydrolyses are presented in Table 3, and the time courses are provided in the supplementary information (Additional file 1). After 48 h of hydrolysis, 16% and 29% of the glucan were converted to glucose when hydrolyzed with 10 and 20 FPU/g cellulose, respectively. These yields are significantly lower than previously reported values (40─70%), which were achieved under similar conditions with HEX-pretreated birch wood [43]. The authors attributed the high conversion yields to effective removal of lignin during HEX, particularly from the fiber surface [43]. Due to the similarity of HEX conditions applied and the compositions of the treated materials, significant differences in lignin surface coverage and topochemistry are unlikely explanations for the discrepancy in hydrolyzability. It is more likely to be attributed to the different raw materials used—specifically the lower S/G ratio of the lignin in the birch–beech wood mixture compared with birch alone [42]. G lignin has a higher cellulase adsorption affinity than S lignin, which increases the unproductive binding of cellulases to lignin [56]. Other variations in the experimental setup, such as particle size and aspect ratio, enzyme cocktail, and incubation conditions, have been shown to affect the enzymatic hydrolysis of lignocellulosic materials [57–59] and might have accounted for the disparity in hydrolyzability.

**Table 3:** Conversion yields after 48 h and initial rates of the enzymatic hydrolysis of SF_STEX_, SF_HEX150/8_, SF_STEX+HEX150/8_, and SF_STEX+HEX190/4_

	Conversion yield^a^ (%)		Initial rate^b^ (g/L/h)	
	10 FPU/g^c^	20 FPU/g^c^	10 FPU/g^c^	20 FPU/g^c^
SF_STEX_	40 ± 2	75 ± 3	0.40 ± 0.04	0.80 ± 0.01
SF_STEX+HEX150/8_	26 ± 1	47 ± 2	0.51 ± 0.03	0.88 ± 0.07
SF_STEX+HEX190/4_	24 ± 3	39 ± 6	0.39 ± 0.03	0.72 ± 0.04
SF_HEX150/8_	16 ± 0	29 ± 0	0.24 ± 0.00	0.38 ± 0.01

*SF* solid fraction, *STEX* steam explosion, *HEX* hydrotropic extraction

^a^Glucan yield after 48 h of reaction

^b^Analyzed for the first 8 h of reaction

^c^Enzyme loading in FPU/g cellulose

#### The hydrolyzability of SF_STEX_, SF_STEX+HEX150/8_, and SF_STEX+HEX190/4_

**Fig. 3:**
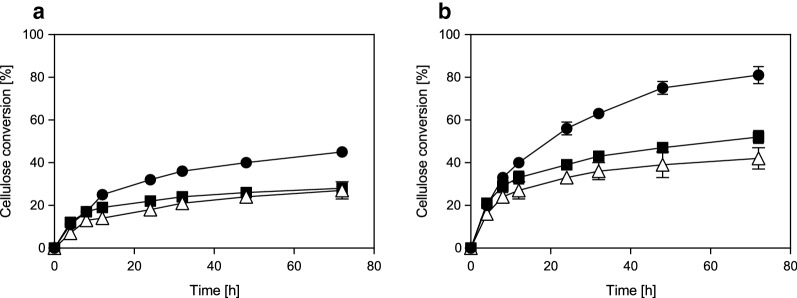
Hydrolyzability of the pretreated materials. The conversion efficiencies of SF_STEX_ (filled circles), SF_STEX+HEX150/8_ (filled squares), and SF_STEX+HEX190/4_ (empty triangles) are shown. Hydrolysis was conducted with 10 FPU/g cellulose (panel a) and 20 FPU/g cellulose (panel b) as enzyme loadings. The data represent the mean values of four experiments. Error bars indicate the standard deviation

In the next step, the glucan-enriched solid from the STEX and combined processes (SF_STEX_, SF_STEX+HEX150/8_, and SF_STEX+HEX190/4_) were hydrolyzed enzymatically with enzyme loadings of 10 and 20 FPU/g cellulose. The resulting time courses are presented in Fig. 3, and the conversion yields and initial rates are summarized in Table 3. The hydrolyzability of SF_STEX+HEX150/8_ and SF_STEX+HEX190/4_ exceeded that of SF_HEX_, resulting in ~ 1.6- and ~ 1.4-fold increases in hydrolyzability, respectively. However, SF_STEX_ was hydrolyzed significantly more efficiently than SF_STEX+HEX150/8_ and SF_STEX+HEX190/4_. The yields after 48 h for SF_STEX+HEX150/8_ and SF_STEX+HEX190/4_ were only ~ 63–-65% and ~ 52–60% of that for SF_STEX_ (Table 3). SF_STEX+HEX150/8_ and SF_STEX+HEX190/4_ contained significantly less lignin (Table 2) and a similar (SF_STEX+HEX150/8_) or lower (SF_STEX+HEX190/4_) xylan content than SF_STEX_. Because the removal of hemicellulose and the breakdown of the LCC shield has been described to improve hydrolyzability [58–61], this result was unexpected.

**Fig. 4:**
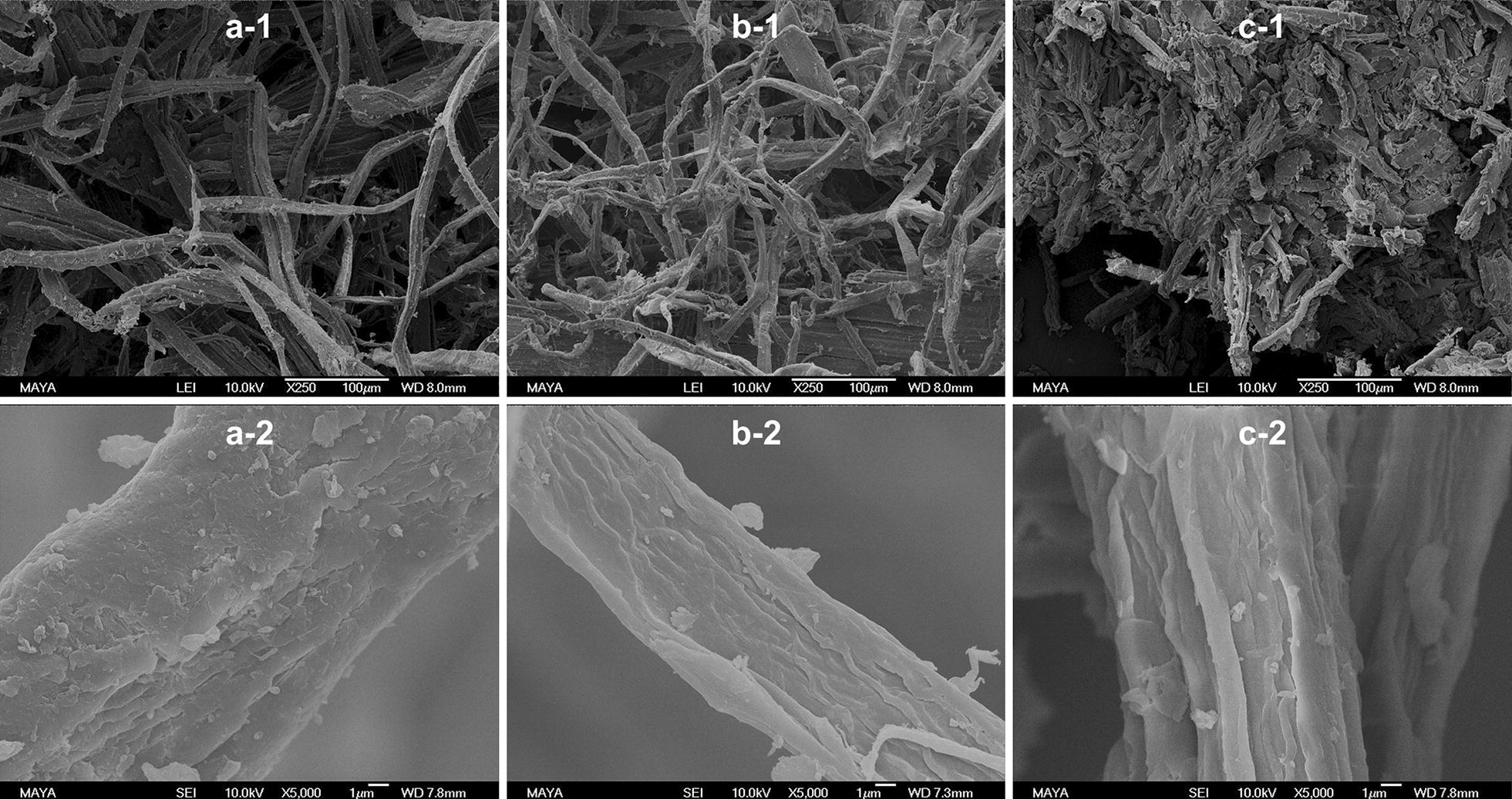
SEM images of SF_STEX_ (panels a1/2), SF_STEX+HEX150/8_ (panels b1/2), and SF_STEX+HEX190/4_ (panels c1/2). The magnification was 250- (panels a/b/c-1) and 5000-fold (panel a/b/c-2)

To better understand the structural changes in the materials caused by the different pretreatments, scanning electron microscopy (SEM) was performed. The resulting images are shown in Fig. 4. Compared with SF_STEX_ (Fig. 4a-1), SF_STEX+HEX150/8_ had finer fibers (Fig. 4b-1). The fiber surface was rougher, and single cellulose fibrils were exposed (Figs. 4a-2, 4b-2), indicating the removal or redistribution of surface lignin and hemicellulose [30, 43]. Further increase in temperature during HEX resulted in the complete collapse of the fibrillose structure (SF_STEX+HEX190/4_, Fig. 4c-1), and further removal of the LCC matrix that embedded the cellulose fibers was observed (Fig. 4c-2). Exposure of the cellulose fibers and decreases in particle size and aspect ratio have been described to increase accessibility and thus the hydrolyzability [57, 58, 61]. Thus, SEM imaging cannot explain the decline in hydrolyzability for SF_STEX+HEX150/8_ and SF_STEX+HEX190/4_ compared with SF_STEX_.

#### Productive versus non-productive enzyme binding to SF_STEX_, SF_STEX+HEX150/8_, and SF_STEX+HEX190/4_

**Fig. 5:**
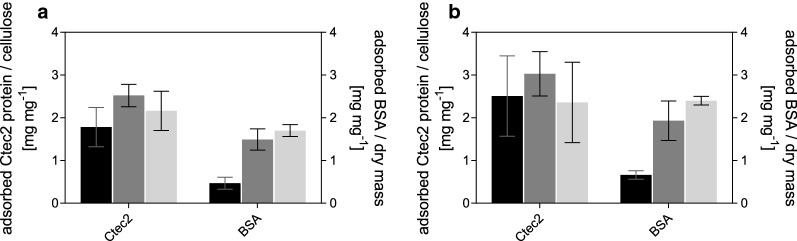
Specific and nonspecific adsorption of proteins onto the pretreated materials. The ratios of adsorbed proteins to the loaded cellulose (Cellic Ctec2) and dry mass (BSA) for SF_STEX_ (black bars), SF_STEX+HEX150/8_ (dark grey bars), and SF_STEX+HEX190/4_ (light grey bars) are shown. The protein loadings were equivalent to 10 FPU/g cellulose (panel a) and 20 FPU/g cellulose (panel b). The data represent the mean values of 4 experiments. Error bars indicate the standard deviation

The ability of enzymes to access their binding sites is one of the main factors influencing biomass hydrolyzability [57, 58, 61]. Total protein adsorption is the sum of enzymes and proteins productively and non-productively bound to the carbohydrate structures [62, 63] and those adsorbed onto the lignin [17, 59, 60]. To evaluate whether the substrates show differences in binding enzymes and to estimate the amount of protein that is lost due to adsorption onto lignin, a protein adsorption study with Cellic Ctec2 and BSA was performed. The results are summarized in Fig. 5.

Independent of the enzyme loading, more Cellic Ctec2 proteins were adsorbed onto the cellulose of SF_STEX+HEX150/8_ than to SF_STEX_. Although these results are within the standard deviation, the increase was likely caused by the lower lignin content of the SF_STEX+HEX150/8_, which can enhance the cellulose accessibility to enzymes by reducing the steric hindrance of the lignin [62]. This is supported by the rates calculated for the initial hydrolysis reaction, which were up to 1.3-fold higher for SF_STEX+HEX150/8_ than for SF_STEX_ (Table 3). After 8 h, however, the reaction slowed down more drastically for SF_STEX+HEX150/8_ (Fig. 3), signifying that a factor other than cellulose accessibility was responsible for the observed hydrolysis profiles.

When BSA was used, SF_STEX+HEX150/8_ adsorbed substantially more protein than SF_STEX_ (Fig. 5). This suggests that the combined pretreatment results in a lignin with increased tendency to hydrophobically interact with the proteins [17, 59, 60]. Under the conditions for STEX and HEX, lignin cycles between the solid and liquid phases in a series of phase transitions, solubilization events, and reactions, resulting in its chemical modification and redistribution [14, 18, 59]. The resulting condensed lignin can increase the adsorption of proteins substantially [17, 60], and as a result, more enzymes were immobilized over hydrolysis time to the lignin of SF_STEX+HEX150/8_ than to that of SF_STEX_ [17, 60]. This reduction of active enzyme over reaction time might well explain the lower hydrolysis yields (Table 3, Fig. 3).

When comparing SF_STEX+HEX150/8_ and SF_STEX+HEX190/4,_ a decrease of Cellic Cetc2 protein adsorption to cellulose and a slight increase in BSA adsorption was observed (Fig. 5). The initial rates and yields were lower for SF_STEX+HEX190/4_ than for SF_STEX+HEX150/8_ (Table 3). Due to the parity of the lignin content of these two materials (Table 2), as well as the relatively small variation in BSA binding, it seems unlikely that the lignin caused observed differences in the rates and yields. Based on SEM imaging (Fig. 4c), the underlying reason for the observed discrepancy could be a reduced accessibility of cellulose to enzymes caused by the collapse of the fibril structure [62, 63]. Because this cannot be elucidated with the present experimental setup, we will perform a more detailed analysis of the cellulose accessibility to cellulases in the near future, i.e., by inactivated enzymes or carbohydrate binding modules.

## Conclusion

In this work, we present a sequential approach to fractionate and isolate lignocellulosic components in hardwoods for further valorization. The two-step process successfully demonstrated the selective solubilization of hemicellulose and lignin in sequential steps, producing aqueous hemicellulose and lignin product streams with 73 wt% and 50 wt% yield, respectively. Simultaneously, the presented approach produced solids that were nearly twofold enriched in cellulose, at 99 wt% cellulose recovery. The three resulting product streams provide versatile intermediate platforms for biological and catalytic upgrading to renewable bio-based fuels, chemicals, and materials. In a broader context, this work provides an alternative for biomass fractionation with proven scalability that is more environmentally benign than conventional technologies, paving way for the evolving integrated forest biorefinery.

## Additional file

**Additional file 1.** Enzymatic hydrolysis (panel a) and protein adsorption analysis (panel b) of materials pretreated with HEX alone (SF_HEX150/8_). The conversion efficiencies of SF_HEX150/8_ with 10 FPU/g cellulose (filled circles) and 20 FPU/g cellulose (empty circles) are shown. The adsorption was analyzed with Cellic Ctec2 and BSA, and the protein loads were equivalent to 10 FPU/g cellulose (black bars) and 20 FPU/g cellulose (grey bars). The data represent the mean values of 2 experiments. Error bars indicate the spread.

## Abbreviations

BSA: bovine serum albumin
DM: dry matter
FPU: filter paper unit
G: guaiacyl
HEX: hydrotropic extraction
HPLC: high-performance liquid chromatography
LCC: lignin–carbohydrate complex
MHC: minimum hydrotrope concentration
S: syringyl
SEM: scanning electron microscopy
STEX: steam explosion
SXS: sodium xylene sulfonate
