# Supplementary figures and images for: Sequential fractionation of the lignocellulosic components in hardwood based on steam explosion and hydrotropic extraction
Source: Biotechnol Biofuels. 2019 Jan 4;12:1. doi: 10.1186/s13068-018-1346-y (PMC6318938; doi:10.1186/s13068-018-1346-y)

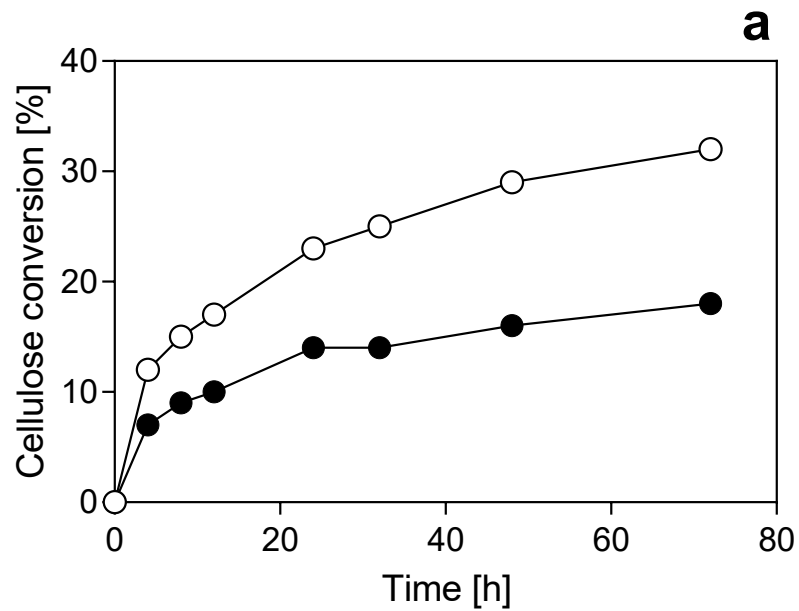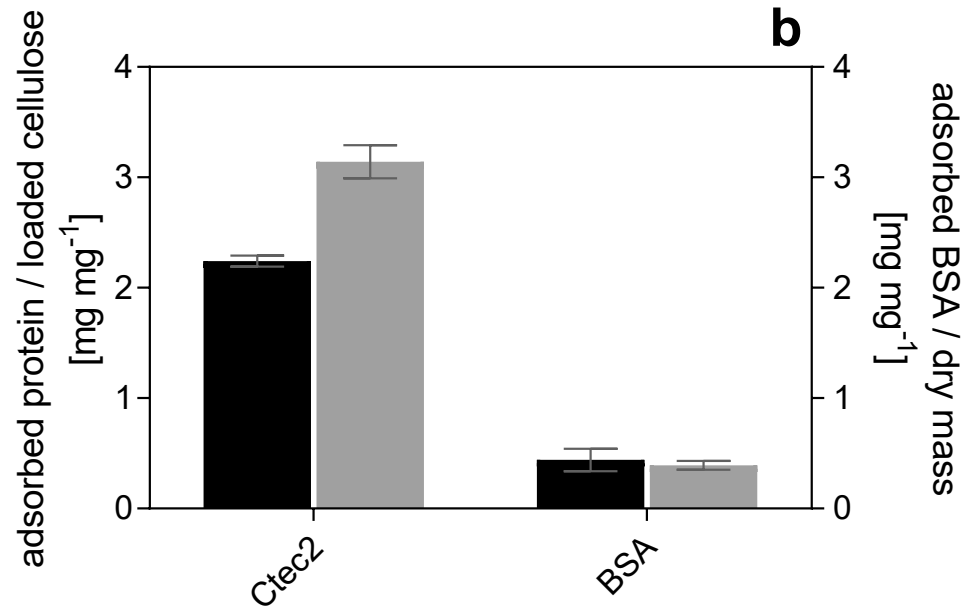

Supplement: Supplementary file 1 — Additional file 1. Enzymatic hydrolysis (panel a) and protein adsorption analysis (panel b) of materials pretreated with HEX alone (SFHEX150/8). The conversion efficiencies of SFHEX150/8 with 10 FPU/g cellulose (filled circles) and 20 FPU/g cellulose (empty circles) are shown. The adsorption was analyzed with Cellic Ctec2 and BSA, and the protein loads were equivalent to 10 FPU/g cellulose (black bars) and 20 FPU/g cellulose (grey bars). The data represent the mean values of 2 experiments. Error bars indicate the spread. [file 13068_2018_1346_MOESM1_ESM.pdf]
